# Clinical acupuncture therapy for femur head necrosis: A protocol for systematic review and meta analysis

**DOI:** 10.1097/MD.0000000000026400

**Published:** 2021-06-25

**Authors:** Hongyu Wang, Fengyun Yang, Zhiwen Cao, Yunfeng Luo, Jiangyuan Liu, Zhijun Yang, Hanting Xia, Fuwei Li, Zhaochong Mao, Wenlong Yang

**Affiliations:** a Jiangxi University of Traditional Chinese Medicine; b The Affiliated Hospital of Jiangxi University of Traditional Chinese Medicine, Nanchang, China.

**Keywords:** acupuncture, Femur Head Necrosis, protocol, systematic review and meta-analysis

## Abstract

**Background:**

Femur Head Necrosis (FHN) is a common clinical joint orthopedic-related disease, and its incidence is increasing year by year. Symptoms include dull pain and dull pain in the affected hip joint or its surrounding joints. More severely, it can lead to limited joint movement and inability to walk autonomously. Surgical treatment has many sequelae. The high cost makes it unaffordable for patients, and the side effects of drug treatment are unknown. A large number of clinical studies have shown that acupuncture is effective in treating femoral head necrosis. Therefore, this systematic review aims to explore the safety and effectiveness of acupuncture in the treatment of femoral head necrosis.

**Methods:**

We will conduct a comprehensive literature search in Medline, PubMed, Cochrane Database of Systematic Reviews, Embase, Chinese Biomedical Literatures Database (CBM), China National Knowledge Infrastructure (CNKI), Wang FangDatabase (WF), Chinese Scientific Journal Database (VIP) from inception to May 2021 without any language restriction. In addition, we will retrieve the unpublished studies and the references of initially included literature manually. The two reviewers will identify studies, extract data, and assess the quality independently. The outcomes of interest include: total effective rate; the total nasal symptom score; Hip function (Hip Harris joint score, WOMAC hip score, hip joint Lequesne index score, Merle D ’Aubigne and hip joint Postel score); Adverse events. Randomized clinical trials will be collected, methodological quality will be evaluated using the Cochrane risk-of-bias assessment tool, and the level of evidence will be rated using the Grading of Recommendations, Assessment, Development and Evaluation approach. Meta-analysis will be performed using RevMan 5.4.0 software. The heterogeneity test will be conducted between the studies, *P* < .1 and I^2^ > 50% are the thresholds for the tests. We will utilize the fixed effects model or the random effects model according to the size of heterogeneity.

**Results:**

The meta-analysis program will systematically evaluate the efficacy and safety of acupuncture in the treatment of FHN patients.

**Conclusion:**

This study will investigate whether acupuncture can be used as one of the non-surgical and non-pharmacological therapies for the prevention or treatment of FHN.

**Trial registration number:**

INPLASY202150035.

## 1 Introduction

Femur Head Necrosis (FHN) is a potentially devastating disease,^[1]^ characterized by the death of bone cells and bone marrow, mainly caused by insufficient blood supply to the subchondral bone.^[2]^

FHN is clinically manifested as pain in the affected hip. The nature of the pain is described by most patients as a deep, intermittent, paroxysmal pain. The pain is usually limited to the groin area, or involves large and small tuberosity areas, and the pain is radioactive, radiates to the hip and knee on the same side, weight-bearing and aggravation after activities, in more serious cases will lead to limited joint activities.^[2,3]^

The occurrence of FHN is related to many factors, but the underlying specific pathogenesis is unclear.^[4]^ Recent animal experiments have shown that glucocorticoids can reduce the expression of β-catenin and c-Myc downstream of the Wnt pathway, leading to the apoptosis of bone cells and osteoblasts, which in turn causes early hormonal FHN.^[5]^ DKK1 is a Wnt pathway inhibitor that can regulate the development of bone tissue. Alcoholism promotes the expression of DKK1, which in turn causes a large number of bone cell apoptosis and promotes the progression of FHN.^[6]^ In addition to the effects of hormones and alcohol abuse, blood flow disorders of the femoral head,^[7,8]^ lipid metabolism disorders,^[9]^ increased intra-articular pressure,^[10]^ osteoblast apoptosis,^[11,12]^ gene specific risk factors such as polymorphism,^[13]^ family history of FHN^[14]^and other related diseases can affect microcirculation in some way.^[15]^ Domestic and foreign scholars have done a lot of research on the pathogenesis of FHN, but no breakthrough has been made so far. But what we knew is that the common developmental endpoint of diseases are caused major risk factors such as abnormal microcirculation and necrosis, and subsequent collapse of subchondral bone, eventually leads to the occurrence of FHN.

FHN can be seen at any age, and there is no gender difference.^[16]^ Globally, FHN has a high probability of disability, which seriously affects people's health and quality of life.^[17,18]^ In the United States, there are about 20,000 new FHN patients every year.^[19]^ In the United Kingdom, the average age of onset of FHN is 58.3 years, and the prevalence rate is 2 out of every 100,000 people with this disease.^[17]^ In German-speaking countries, the incidence of FHN is 0.01%, and 5000 to 7000 people suffer from this disease every year.^[20]^ Japanese scholars Ikeuchi K, Hasegawa Y found that the annual incidence of non-traumatic FHN in Japan is about 1.9 per 100,000.^[21]^ In addition, in China, an epidemiological study found that in the general population, about 8.2 million Chinese people over the age of 15 are affected by the disease.^[22]^ If FHN is not treated in time, 90% of patients will experience femoral head collapse within 5 years, and then joint dysfunction will eventually lead to paralysis.^[23]^

Based on the characteristics of FHN patients with high harm, high morbidity, poor prognosis, femoral head collapse and hip joint destruction at the end of the disease, most doctors will recommend that patients undergo surgical intervention, that is, the characteristics of artificial total hip arthroplasty (THA), but THA surgery has certain adverse effects.^[16]^ Moreover, the cost of THA is prohibitive. In recent years, studies have shown that the cost of THA in China is between 8000 and 10,000 US dollars,^[22]^ which brings both patients and the country posed a huge economic and social burden. At present, there are generally two non-surgical treatment methods: physical therapy and drug therapy. Physical therapy includes hyperbaric oxygen (HBO)^[24]^ and pulsed electromagnetic field (PRMF).^[25]^ HBO has significant effects but requires continuous treatment. The course of treatment is longer, PRMF can only slightly delay the process of FHN, and the course of treatment is not significant. Relevant drug treatment: Western medicine treatment generally uses statin lipid-lowering drugs, alendronate sodium, etc., statin lipid-lowering drugs have a significant effect on hormone-induced FHN,^[26]^ but for FHN caused by other reasons, its efficacy is unknown. Alendronate has a significant effect on early FHN,^[27]^ but it has no significant effect on late disease.^[28]^ Recent studies have shown that: Chinese medicine has its unique advantages in the treatment of this disease.^[29]^ FHM treats four types of phlegm and blood stasis blocking the collaterals, qi stagnation and blood stasis, obstruction of meridians, and liver and kidney deficiency.^[30]^ The main treatment methods include dredging collaterals and removing numbness, activating blood stasis, replenishing liver and kidney. Zhiguo Liu^[31]^ et al have verified through animal experiments that epimedium and core decompression can reduce blood rheology indexes, improve local microcirculation around joints, promote bone growth, accelerate the repair of damaged tissues and has a good effect. Liao Hongwei^[32]^ et al. used Fuyang Huogu Pills combined with core decompression to treat 68 patients with early FHN. Follow-up showed that this method can greatly reduce the probability of patients undergoing THA. However, there is no direct evidence to prove that there are single Chinese medicines or prescriptions that have a significant effect on FHN, and both require core decompression therapy or THA surgical intervention. Therefore, FHN patients need a safe and effective alternative therapy to alleviate the pain caused by the disease.

Acupuncture therapy is one of them. This therapy is currently commonly used in combination with drugs and core decompression to treat FHN-induced pain around the hip joint, and the effect is remarkable, and it is gradually accepted and used.^[33,34]^ Some studies have found that the mechanism of acupuncture treatment of FHN may be related to the improvement of peripheral blood TNF-α and VEGF levels.^[35]^ Of course, some studies have shown that acupuncture combined with drug treatment can improve the hemodynamics of patients, improve hip joint function, reduce the volume of necrotic area, increase the gray scale of osteonecrotic area and femoral head.^[36]^

In addition, it originated from traditional Chinese medicine (TCM)'s thinking that “treatment is as good as treatment itself” and that “health is maintained and not harmful to others”. Acupuncture therapy has the characteristics of regulating qi, blood, yin and yang, preventing diseases, having fewer side effects, and being easily accepted by patients. Therefore, it has gradually become popular around the world.^[37,38]^

Although the effects of acupuncture and moxibustion have been widely confirmed, the effectiveness and safety of acupuncture in the treatment of FHN are still controversial. System review or Meta analysis has been considered as the basis for evaluating clinical efficacy and formulating clinical guidelines. Therefore, this study adopts the method of evidence-based medicine to systematically analyze and evaluate randomized controlled trials (RCTs) of acupuncture and moxibustion treatment of patients with FHN to provide a basis for verifying the efficacy.

System analysis helps to evaluate the effectiveness and credibility of clinical methods.^[39]^ So far, the number of patients with femoral head necrosis has increased, and the simplicity and low side effects of acupuncture therapy have attracted the attention of clinicians at home and abroad. Randomized controlled trials (RCT) of acupuncture treatment of femoral head necrosis have also increased simultaneously. At present, there is still a lack of relevant systematic reviews in clinical practice. Therefore, this systematic review focuses on the safety and effectiveness of acupuncture in the treatment of femoral head necrosis, in order to cope with patients who cannot afford high surgical costs, adverse drug reactions or who wish to receive acupuncture treatment, so that they can better serve the clinic.

## 2 Methods and analysis

### 2.1 Study registration

This system evaluation plan has been registered on the International Prospective Register of Systematic Reviews (registration number INPLASY202150035). This webpage (https://inplasy.com/inplasy-2021–5–0035) can be used to verify its legitimacy. This article does not require ethical approval because it simply examines FHN acupuncture treatment that has been documented in multiple databases. The protocol is based on the Cochrane Handbook for Systematic Reviews and Meta-Analysis Protocol (Cochrane Handbook for Systematic Reviews and Meta-Analysis Protocol) (PRISMA-P).^[40]^

### 2.2 Eligibility criteria

#### 2.2.1 Type of studies

We will comprehensively search the literature on acupuncture treatment in FHN patients in Chinese and English databases. In addition, unpublished documents will be searched manually. The non-randomized controlled trials must be excluded.

#### 2.2.2 Types of participants

Patients diagnosed with necrosis of femoral head according to history, symptoms, signs and imaging examination. Age, sex and origin of the patients are not restricted.

#### 2.2.3 Types of interventions

The FHN in the experimental group in the test group must be treated with acupuncture as the main regimen (either in combination with other treatments or alone) and the control group must be treated with non-acupuncture therapy.

#### 2.2.4 Type of comparators

Massage, medicine (Traditional Chinese medicine, western medicine), routine symptomatic therapy, and other intervention methods may be utilized in the control group, but acupuncture should not be employed as the sole requirement.

1. Acupuncture therapy vs no treatment;
2. Acupuncture therapy vs placebo;
3. Acupuncture therapy vs sham acupuncture;
4. Acupuncture therapy vs symptomatic or active treatment;

#### 2.2.5 Types of outcome measures

##### 2.2.5.1 Primary outcomes

Both the total effective rate and hip function (Harris score, WOMAC score, etc.) were the primary outcomes. The total score of nasal symptoms will be scored according to the patient's disease history, symptoms, signs and imaging.

##### 2.2.5.2 Secondary outcomes

1. Hip function (Hip Harris joint score, WOMAC hip score, hip joint Lequesne index score, Merle D ’Aubigne and hip joint Postel score);
2. Hip pain score (visual analogue Scale, VAS score);
3. Progress of imaging staging of femoral head necrosis Rate (staging method: ARCO, FICAT, Japanese Joint Association Staging);
4. volume percentage of femoral head necrosis;
5. Total hip replacement rate.^[41]^

### 2.3 Exclusion criteria

Before and after therapy; Acupuncture is present in the control group; Repeated literature, theoretical discussion and review literature, nursing literature, animal experimental research, etc.

### 2.4 Search strategy

We will search the following databases: PubMed, Cochrane Database of Systematic Reviews, Embase, Chinese Biomedical Literatures Database (CBM), China National Knowledge Infrastructure (CNKI), Wang FangDatabase (WF), Chinese Scientific Journal Database (VIP) from their inception to May 2021. The main subject terms searched: “acupuncture”, “femur head necrosis”, “osteonecrosis of femoral head”, “avascular necrosis of femur head”, “ANFH”, “ONFH”, Pubmed's search strategy is shown in Table 1; Other database search strategies will be adjusted according to each database.

**Table 1:** The search strategy for Pubmed.

Order	Strategy
#1	Search “femur head necrosis”[Mesh]
#2	Search “osteonecrosis of femoral head” [Title/Abstract] or “femoral head necrosis” [Title/Abstract] or “femur head necroses” [Title/Abstract] or “aseptic necrosis of femur head” [Title/Abstract] or “ischemic necrosis of femoral head “ [Title/Abstract] or “avascular necrosis of femur head” [Title/Abstract] or “ONFH” [Title/Abstract]or “ANFH” [Title/Abstract]
#3	#1 OR #2
#4	Search “Acupuncture” [Mesh] OR “Acupuncture Therapy” [Mesh] OR “Acupuncture, Ear” [Mesh] OR “Acupuncture Points” [Mesh]
#5	Search “acup^∗^” [Title/Abstract] or “need^∗^” [Title/Abstract] or “Acupuncture Therapy” [Title/Abstract] or “Acupuncture treatment “ [Title/Abstract] “Pharmacopuncture” [Title/Abstract] or “Meridians” [Title/Abstract] or “ Acupuncture Points” [Title/Abstract] or “electropuncture “ [Title/Abstract] “ Ear acupuncture” or ”Fire needle ” [Title/Abstract] or ”Warming needle” [Title/Abstract]
#6	#4 OR#5
#7	Search “Randomized controlled trial” [MeSH] or “controlled clinical trial” [MeSH]
#8	Search “Randomized controlled trial” [Title/Abstract] or “clinical trial” [Title/Abstract] or “randomized” [Title/Abstract]
#9	#7 OR #8
#10	#3AND#6AND#9

### 2.5 Process of selection

**Figure 1:**
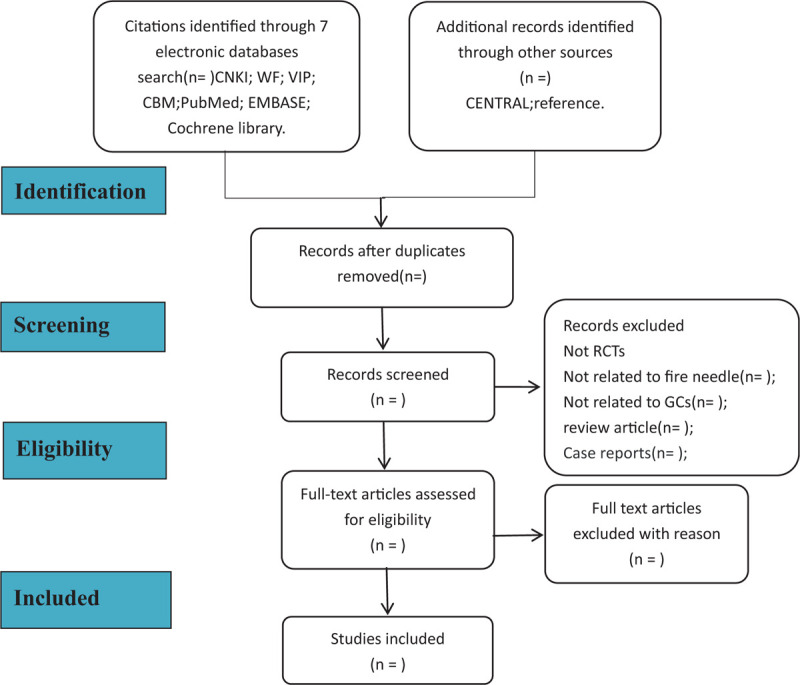
Flowchart of literature selection.

We will deal with the included literature in the following way and the specific operation is as follows: Firstly, according to the selected topic, the retrieved literature is imported into noteExpress 3.0 in the document manager in the correct retrieval way, and remove the duplicate published article in the document manager; Then, by reading the title and abstract one by one, the article irrelevant to this research will be weeded; then, the remaining articles will be downloaded in sequence and the full text will be read; finally, based on the inclusion and exclusion criteria required in this article, the final paper will be framed;

For this operation, 2 researchers (Yunfeng Luo, Zhijun Yang, Hanting Xia) strictly follow this procedure. If they disagree, consult the third evaluator (Hongyu Wang) for negotiation. The included article process is shown in Figure 1.

#### 2.5.1 Information extraction

In the final article, two researchers (Yunfeng Luo, Zhijun Yang, Hanting Xia) independently extracted information and entered it into word 2010. After the end, they were cross-checked. If there are any doubts, they can contact the third evaluator (Hongyu Wang) Negotiate processing to ensure the accuracy of the information. The extracted information includes: title, Author, year of publication, sample size, intervention measures and treatment course, etc. When some important information is missing in the input data of some articles, contact the author by phone or email.

#### 2.5.2 Methodological quality evaluation

Use the Cochrane Reviewer's Handbook 5.0 to evaluate the quality of the final selected literature and assess the risk of bias.^[42]^ The main contents are: Random method; allocation concealment; implementation of blind method; blindness of the outcome rater; completeness of the result data; selective reporting of results; other biases. Each of the above three items contains “yes”, “no” and “unclear”. The two assessors (Yunfeng Luo, Zhijun Yang, Hanting Xia) need to evaluate the options that meet the conditions. If there is any dispute among them, they can discuss and deal with a third party (Hongyu Wang).

### 2.6 Data synthesis

#### 2.6.1 Quantitative data analysis

Enumeration data will be represented by odds ratio (OR) and 95% confidence interval (CI), measurement data are represented by weighted mean difference (WMD) and 95% confidence interval (CI) or standardized mean difference (SMD) should be used when the units were not unified.

#### 2.6.2 Heterogeneity analysis

When performing heterogeneity test, use I^2^ test. When *P* > .1 and I^2^<50%, use fixed effects model; Otherwise, use random effects model. Sensitivity analysis will be used if the heterogeneity is large. If there is substantial heterogeneity, it can be analyzed descriptively. Use Review Manager 5.4.0 line inverted funnel chart to qualitatively analyze publication bias.

#### 2.6.3 The publication bias

If the number of remaining articles is more than 10, we can use Review Manager 5.4.0 line inverted funnel chart to qualitatively analyze the publication bias. The graph shows the approximate shape which represents the publication bias.

#### 2.6.4 Subgroup analyses

If there is large heterogeneity, we will conduct subgroup analysis based on different control measures.

#### 2.6.5 Sensitivity analysis

The sensitivity analysis should be performed to assess the reliability of the meta-analysis, Analysis software uses STATA 14.0 software for sensitivity analysis.

## 3 Discussion

This review is the first of the current modern literature on the treatment of FHN by acupuncture and moxibustion. The purpose of this review is to find evidence of the benefit of acupuncture and moxibustion in the treatment of FHN. Based on the discussion part of this paper, following aspects are presented in detail:

1. Study on TCM pathogenesis of FHN
2. Advantage, disadvantages and mechanisms of acupuncture and moxibustion treatment of FHN
3. Horizontal comparison with other treatment methods or viewpoints;
4. Explain the results;

## 4 Conclusion

Acupuncture has a history of thousands of years and has been used by doctors at home and abroad as a complementary and alternative therapy. The use of acupuncture as a non-surgical treatment is gradually spreading. Acupuncture treatment has become the dominant disease of hip pain and other diseases, and intervention may play a role by regulating a variety of pathways and activating a variety of medium.^[43,44]^ Currently, there is no systematic and scientific evaluation of acupuncture for FHN, so this paper aims to provide an evidence-based medical perspective on the safety of acupuncture for FHN. Of course, this study still has some limitations: First, the quality of the article is not high, which may affect the evaluation results; Second, the paper is limited in Chinese and English language, which may lead to incomplete literature retrieval; Third, contacting the article's author may be impossible, resulting in inadequate data results. As a result, more high-quality randomized controlled trials and research procedures are needed to demonstrate its efficacy, allowing for a more objective assessment of acupuncture's safety and efficacy in FHN.

## Author contributions

**Conceptualization:** Hongyu Wang.

**Data curation:** Fengyun Yang.

**Formal analysis:** Hongyu Wang, Zhiwen Cao.

**Methodology:** Jiangyuan Liu.

**Software:** Yunfeng Luo.

**Supervision:** Wenlong Yang.

**Validation:** Wenlong Yang.

**Writing – original draft:** Zhijun Yang, Fuwei Li.

**Writing – review & editing:** Zhijun Yang, Hanting Xia, Zhaochong Mao.
